# Reviews of Books

**Published:** 1922-01

**Authors:** 


### REVIEWS OF BOOKS.

**THE PHARMACOPŒIA OF ST. BARTHOLOMEW'S HOSPITAL.** 1921 edition. Edited by J. LANGFORD MOORE, F.C.S. (Spottiswoode, Ballantyne & Co., Ltd.)

Some years ago when Moses was directed to make "a confection after the art of the apothecary" we imagine that the prescription would have been more exact had there been available a copy of the excellent "Pharmacopœia of St. Batholomew's Hospital," a new edition of which has just been issued under the capable editorship of Mr. Longford Moore.

Our memory carries us back to the day when with judicial solemnity Morris handed us a packet of literature of which by far the most useful part was a Bart.'s Hospital Pharmacopœia. But our copy was a chrysalis. This resplendent image now published bears little resemblance to the book we received some sixteen years ago.

The formulæ being alphabetically arranged are easy to find, and these formulæ, mainly used in the special departments, have been placed in separate sections of the Pharmacopœia. The doses are given in both imperial and metric measure, and a table of poisons, with symptoms and treatment of poisoning, has been added.

We have nothing but good to say of this valuable pocket edition, which is a credit to the institution founded by Rahere.—H. J. C.

**BOWEL DISEASES IN THE TROPICS.** By Lieut.-Col. Sir LEONARD ROGERS, C.I.E., F.R.C.P., F.R.S. (Hodder & Stoughton; 30s. net.)

India teems with problems for the medical enthusiast, be they problems of etiology, pathology, treatment or prevention; and many are the illustrious minds that have taken advantage of the possibiliti s there presented. On the side of the masterly control of disease by therapeusis Sir Leonard Rogers has long taken a pronounced stand, and in this volume before us, which collects and summarises the active experience of a lifetime devotion to medicine, we have the latest authoritative contribution to the scientific treatment of bowel diseases which unfortunately so seriously affect life in the tropics, especially in the East.

Cholera and the dysenteries and liver abscess are dealt with in masterly fashion. The history of cholera epidemics and their lessons is discussed with the glamour of romance and the accuracy of the historian. The epidemiology is treated in a way to make the hygienist long to be in the midst of it; and the pathologist will be satisfied that the author is entitled to be ranked amongst the *cognoscenti* in that department of morbid study. In the consideration of treatment it is conceded that the author is truly *facile princeps*, and we can accept with complaisance, dogma and definition as from the legitimate teacher of vast practical experience.

The rationale of procedure and the technique of operation in treatment deserve the careful study of the physician in this country as well as in the tropics; and the precision, lucidity, and meticulous detail with which they are described should instruct the readiness of action and prompt the native wit necessary to the physician in any emergency. With detail we need not here argue; needless to say, individuals will find best results from the technical *adventitia* which most appeal to their own temperament and co-ordinated musculature. The foundation and guide to the goal is however in this book. ¶ Of actual statistical result we are given some hint in the historical study, but we would gladly see in books on tropical medicine more statistical evidence than is here or elsewhere afforded of the morbid significance of disease, contemporary as well as historical.

On Dysenteries and Liver abscess we are again served with a generous collation from an almost unique therapeutic experience. Of Hill Diarrhœa and Sprue we confess to dissatisfaction. The extent of these diseases amongst European and native peoples is not clearly told; the incidence historically is not plain; their regional distribution is dealt with cursorily, superficially and with a conventional reference to authorities of varying credit, which suggests almost an uncertainty of the author of his own conceptions and experience of these diseases. It is perhaps to be regretted that this volume does not deal with theEntericas which loom so large as a morbific factor in life in the East, an especial dread, as Manson emphasises, to the immigrant European. As a comparative study their inclusion would have been of great value. The book, however, as it is, embraces much; the publishers and printers are to be congratulated on their share in presenting the profession with a work of art.—A. M.

**HYGIENE OF WOMEN AND CHILDREN.** By Dr. JANET E. LANE-CLAYPON. (Oxford Medical Publications, Hodder & Stoughton; 15s. net.)

A book of moderate size, with clear print and many illustrations, written by a well-known medical woman, is bound to be attractive. The author considers that since so much has been done in the promotion of hygiene for the public regarded as a community, emphasis requires to be laid on the personal aspect of the question. Hence this book, written chiefly for health visitors and nurses so that they may not fail to appreciate the *raison d'être* of living in such a way as will best ensure health, and the happiness that health brings. The book cannot fail to be read with interest and profit, for it is clear and well-written, and gives much up-to-date information not generally obtainable in books of this kind, as, for example, the work of Prof. Leonard Hill on fresh air and ventilation, the subject of dust and dirt in the atmosphere, &c. The chapters on clothing and footgear are particularly complete, and the paragraphs on the psychology of dress are sound. The illustrations of insanitary housing conditions add much to the text.

Some of the subjects, however, are dealt with in a more advanced way than many of the readers for whom the book is specially intended would probably appreciate, as, for instance, the chapter on "Bacteria in Milk, Standards of Purity."

Theories, be they ever so good, are not always put into practice, and the book may be considered somewhat deficient in some parts in not stating—as well as suggesting—details of practical applications

**Reviews of Books**—(*cont.*).

for after all hygiene is, or should be, essentially practical.

Dr. Lane Claypon's suggestion for giving "bread pulped in hot milk" to infants of eight or nine months, and "mashed potato" a little later, will not commend itself to many infant welfare workers. She omits to consider the dietary at this age according to whether the child has teeth or no.—S. S. F.

**HYGIENE.** By I. Lane Notter and R. H. Firth. 9th edition. (Longmans, Green & Co. Pp. xii. and 540. 10s. 6d. net.)

We extend a hearty welcome to the ninth edition of this well-known and popular work on the principles of hygiene. Its general plan and scope are so familiar that a minute examination and detailed criticism are unnecessary, while its comprehensive survey renders such impossible within the limits of a brief review. The authors, while retaining the old form of the book, have made considerable alterations and additions in order to bring it up to date. Their efforts in this direction have been directed chiefly to the subjects of dietary, disposal of sewage, and infectious disease and sanitary law. A welcome new chapter deals with child welfare and school hygiene. The book remains a reliable guide and effective passport to the more abstruse works on the many-sided and ever-increasingly important subject of preventive medicine.

When will medical writers cease to harrow the feelings of reviewers by the callous and persistent use of the split infinitive ?—C. J.

**ANNUAL REPORT OF THE PUBLIC HEALTH SERVICE OF THE UNITED STATES.** (Washington: Government Printing Office, 1920.)

Such a publication ought, one would think, to be in several folio volumes, each weightier than that with which they say Dr. Johnson once knocked down a publisher. Actually it runs to 391 pages, large octavo, and is, of course, based on reports from health officers of various States, which vary enormously in sanitary efficiency, as is notorious. It is impossible to review this sort of thing exhaustiv.ly, but we offer our readers a few facts. There were over fifty cases of leprosy on the mainland of U.S.A. last year. When the American army evacuated Siberia it was sent to Manila, being first "de-loused" to prevent its bringing with it typhus from Vladivostok. The Philippines appear still to be ravaged with cholera and smallpox. With regard to plague, cyanide-gas fumigation seems to be preferred, being fatal both to rodents and to their fleas. The last few infected rats are the most dangerous, because, the other preferential hosts having been killed off, their parasites are likely to attach themselves to a human host. It is curious to read of yaws having been brought to Philadelphia from France by a Canadian soldier. Another interesting statement is that in many towns and villages in America 25 per cent. of the inhabitants are infected with malaria. The most important administrative feature is the establishment of what is termed a "purveying service," charged with all duties in connection with purchase and issue of supplies. Three main store-houses are planned, one in the central portion of the United States and one each on the Atlantic and Pacific Coasts. By these means speed and economy in distribution will, it is hoped, be furthered. This report will be very interesting to the medical officer of health of an English area, but extensively rather than intensively.

**A TEXT-BOOK OF NURSING PROCEDURES.** By Anna C. Jamme, R.N. (Macmillan; 7s. 6d. net.)

Briefly, this book is a collection of notes from which to give forty-one demonstrations on everyday hospital procedures, such as the patient's toilet, saline infusion, enemata, hot applications, etc., etc. Clear photographs accompany each demonstration. If one seeks to criticise, the illustrations figs. 49 and 50 show the nurse's left hand in an awkward and unusual position while giving a hypodermic injection, and in demonstration 25, page 82, it is much more satisfactory to take a sterile catheter specimen of urine for the laboratory direct into the sterile flask, instead of pouring the urine from a dish into the flask, as the author directs. Every teaching school has its own methods, and much valuable technique is explained in this book.—M.

**CLINICAL STUDIES FOR NURSES.** By Charlotte A. Aikens. (W. B. Saundere Co.)

The author desires to emphasise the fact that the volume is a compilation, and has been written to give to a nurse in the second and third year of training a connected form of theoretical teaching, which she can assimilate without having to read through pages of books of reference. Miss Aikens, out of her wide experience, smooths a real difficulty in a nurse's educational path. Too often the probationer searches in vain among the books at her disposal for practical points, either medical, surgical, administrative, or hygienic, about which she wishes to obtain information. The spelling in this book is American ; the plates and diagrams are excellent.—M.

**ORTHOPÆDIC APPARATUS.** By F. G. Ernst. Pp. vi., 99, with illustrations. 1921. (The Whitefriars Press, Ltd. 12s. 6d. net.)

This book represents the result of many years' work as an orthopædic mechanician. The appliances therein described are the outcome of co-operation between the surgeon and the mechanic. The suggestions made by the various surgeons have enabled the author to provide a suitable apparatus. The apparatus varies from the very simplest to the most complex, and consequently a very wide field is covered. Few conditions arise for which appliances have not been designed.

The author is Orthopædic Mechanician to the National Orthopædic Hospital, and much of his work has been done in association with the surgeons there. The book is divided into eight sections and starts with the head and neck and works down to the feet. The illustrations consist of 65 plates which contain 193 illustrations, beautifully and clearly depicted, and show apparatus carried to a fine art. The conditions for which appliances are supplied are

**Reviews of Books**—(*cont.*).

well enumerated, and every piece of apparatus described is figured, much of it both on and off the body. The last section deals with surgical boots, and the last illustration shows evening boots and shoes carried to a fine degree of perfection.

Surgeons and orthopædic surgeons who treat conditions too extensive to be cured by surgical procedures will do well to see what can be done by apparatus, thereby making his patient more independent and his life less of a burden.—T. P. N.

**THE SURGERY OF THE PERIPHERAL NERVE INJURIES OF WARFARE.** By Henry Platt, M.S., F.R.C.S. Pp. 49. 12 illustrations. (Bristol: John Wright & Sons, Ltd. 4s. net.)

The above volume embodies the Hunterian Lectures delivered by the author before the Royal College of Surgeons of England on February 7 and 9, 1921. The author's experience has been spread over a period of five years without interruption; during that time he has had an opportunity of observing and treating a very large number of patients suffering from injuries to their peripheral nerves, and has personally performed over 500 operations. He has been associated in his work by a neurological colleague, Professor Stopford. Such co-operation must have been of inestimable value, not only in carrying out clinical and post-operative records, but also as a check in results.

In the earlier work surgeons were of necessity compelled to base operative proceedings on conceptions derived from anatomical, physiological and experimental sources, which at that time seemed to be established as fundamental truths in neurology. The knowledge gained by interim results influenced operative procedures at a comparatively early date. Although these surgical procedures are now mainly retrospective, they are yet to be faced in a certain number of patients.

The work entailed in this volume must have been considerable; the keeping in touch with the pensioner who has been operated on some years ago must be an extremely difficult and trying task. The appendix contains a clear and detailed analysis of the results of suture, and the volume is completed by a fairly extensive bibliography and references.—T. P. N.

**CLINICAL DIAGNOSIS.** By Charles Phillips Emerson. (J. B. Lippincott Co. Fifth edition. 35s. net.)

Ten years have elapsed since the fourth edition of this book was published, and the author has found it necessary to re-write the whole. He is to be complimented upon his discretion in deciding what to leave out and what new matter to insert. A sentence in the preface marks the tone of the whole work: "The one who takes the history of the patient is the only one who can interpret correctly a laboratory finding." Throughout the book the intimate connection which should exist between bedside and bench is emphasised. To the man who is a clinician first and a laboratory-worker second this volume should make a strong appeal. While the practising physician will find all the information of which circumstances will allow him to make practical use, the pure laboratory worker will also find adequate description of the more elaborate methods of examination.

A word of praise is due to the publishers for the way in which this work has been produced; the illustrations are clear and useful and the coloured plates are admirable. A thoroughly useful book—full of information, clearly expressed and free from padding. —C. E. S.

**INTERNATIONAL CLINICS.** Vol. IV. Thirtieth series, 1920. (J. B. Lippincott Co., 2½ dollars per volume. 10 dollars per annum.)

This is one of a well-known series of volumes whose reputation is already secure. They offer to the reader authoritative and instructive articles upon all branches of medicine and surgery, written by acknowledged experts drawn from Great Britain, Europe and America. In the present volume Medicine, Surgery, Paediatrics and Obstetrics are represented. All the chapters are interesting, but mention of a few may be made. A lecture on Tuberculosis by von Pirguet has special reference to the disease in children; he lends the weight of his authority to the view of Ghon that the site of the primary infection is in the lung itself.

In a thoughtful discussion of the Wassermann reaction, Golay of Geneva calls attention to the variability of the reaction in any given case and emphasises the importance of a series of reactions as a guide to prognosis. He advocates strongly the treatment of Syphilis by a combination of Arsenical and Mercurial preparations. Engelbach and Tierney of St. Louis publish an exhaustive study of Pituitary Polyuria, with reports of their own cases and a review of the literature upon the subject; they append a large bibliography which shows unusual care in its compilation and the verification of references. In a useful article upon the Precepts of Infantile Therapeutics Dr. Quintrie-Lamothe of Bordeaux lays stress upon certain general points which, though not new, are apt to be forgotten. Magnuson and Coulter, of Chicago, call attention in a very interesting paper to Sacroiliac strains as a cause of obscure backache and give directions for manipulative treatment that has been successful in their hands. The articles of which this volume is composed vary in value, but all are interesting; the book is well got up, is easy to read and light to hold, and the illustrations are very good. The book is well worth careful reading.—C. E. S.

---

One of our reviewers writes: "I cannot say that I review with real enthusiasm everything that you send to me. Sometimes the stuff is bad, more often it is moderate, and occasionally it is good. Very seldom it is very good indeed, and I wish that you would send me more often material similar to the last—that box of Cadbury's Chocolates. I feel that without much effort I could review them all day long. The only trouble I found in the reviewing is that there was nothing unpleasant to say about them; and, as you know, it is so much easier to write an unkind criticism than the reverse."

